# Toward better assessments of developmental toxicity using stem cell‐based in vitro embryogenesis models

**DOI:** 10.1002/bdr2.1984

**Published:** 2022-01-31

**Authors:** Yusuke Marikawa

**Affiliations:** ^1^ Department of Anatomy, Biochemistry and Physiology Institute for Biogenesis Research, University of Hawaii John A. Burns School of Medicine Honolulu Hawaii USA

**Keywords:** adverse outcome pathway, Daston list, embryo, embryotoxicity, gastruloid, risk assessment, teratogen

## Abstract

In the past few decades, pluripotent stem cells have been explored as nonanimal alternatives to assess the developmental toxicity of chemicals. To date, numerous versions of stem cell‐based assays have been reported that are allegedly effective. Nonetheless, none of the assays has become the gold standard in developmental toxicity assessment. Why? This article discusses several issues in the hope of facilitating the refinement of stem cell assays and their acceptance as the cornerstone in predictive developmental toxicology. Each stem cell assay is built on a limited representation of embryogenesis, so that multiple assays are needed to detect the diverse effects of various chemicals. To validate and compare the strengths and weaknesses of individual assays, standardized lists of reference chemicals should be established. Reference lists should consist of exposures defined by toxicokinetic data, namely maternal plasma concentrations that cause embryonic death or malformations, and also by the effects on the molecular machineries that control embryogenesis. Although not entirely replacing human or animal tests, carefully selected stem cell assays should serve as practical and ethical alternatives to proactively identify chemical exposures that disturb embryogenesis. To achieve this goal, unprecedented levels of coordination and conviction are required among research and regulatory communities.

## 1 INTRODUCTION

Embryogenesis can be disturbed by various chemicals to which the mother is exposed during or before pregnancy. Disturbance during embryogenesis may result in adverse outcomes, namely embryonic death and malformations. To date, a wide range of chemical exposures that cause developmental toxicity have been discovered through human and animal studies. Nonetheless, there are numerous other chemicals, either pharmaceuticals or nonpharmaceuticals, whose developmental toxicity is still unclear. It is a challenging task to determine whether a particular chemical can disturb embryogenesis. Human epidemiologic studies require many cases of miscarriages and birth defects to pinpoint a specific chemical exposure as the culprit. The use of laboratory animals, such as rats and rabbits, allows well‐controlled experiments to assess the effects of chemical exposures during pregnancy. However, high cost, interspecies differences, and animal welfare issues are some of the major drawbacks of animal‐based assays. To ease the burden of human and animal studies and also to facilitate the developmental toxicity screening of many chemicals, nonanimal alternative approaches have been sought out in the past few decades, particularly in vitro assays using pluripotent stem cells.

**FIGURE 1:**
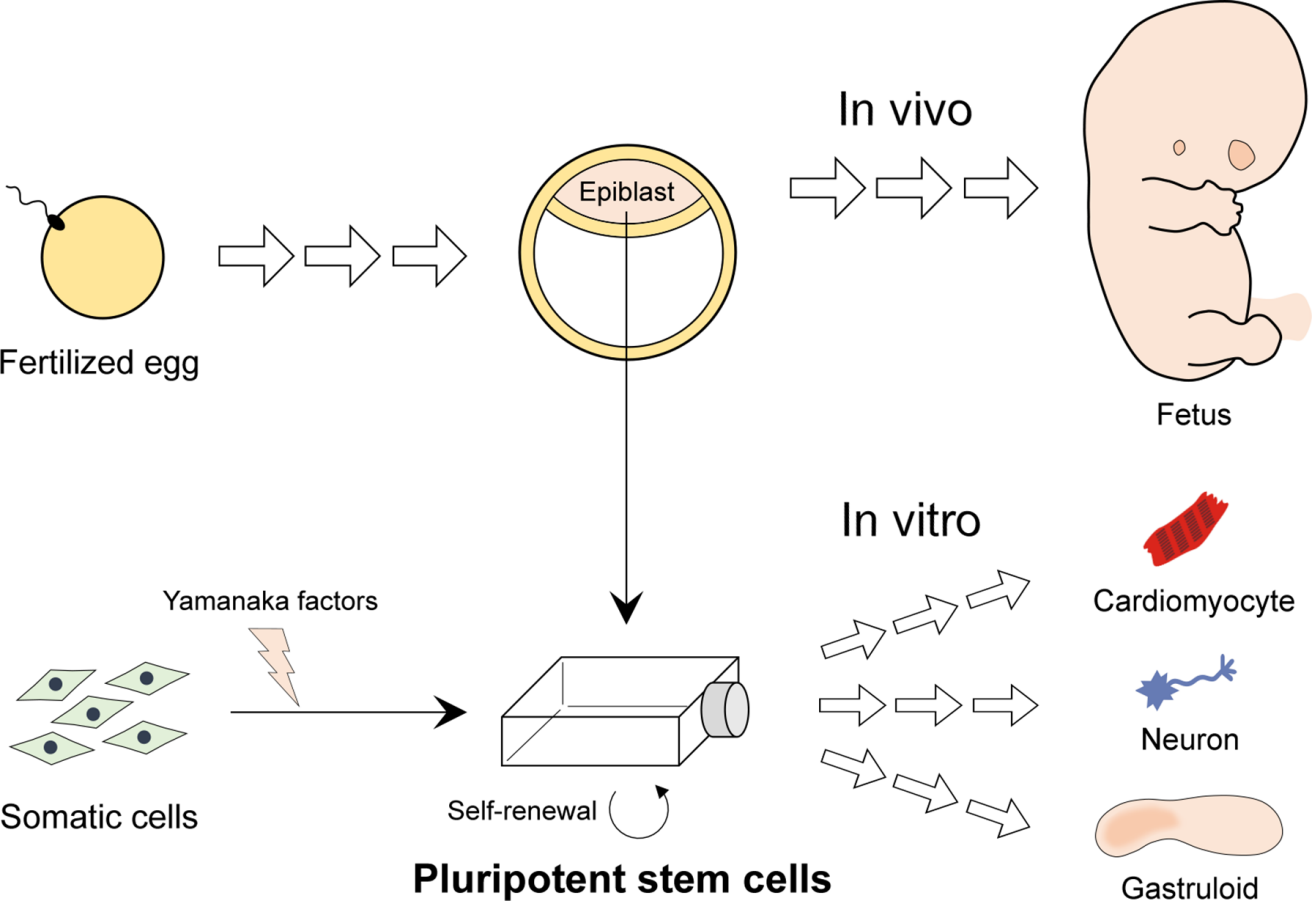
Schematic diagram depicting the origins of pluripotent stem cells: one from the epiblast of normal embryos and the other from somatic cells that are reprogrammed with the Yamanaka factors. In vivo, the epiblast gives rise to the entire fetal body. In vitro, pluripotent stem cells can be induced to differentiate into various cell types, or to form 3D cell aggregates, known as gastruloids, which undergo axial patterning and elongation morphogenesis. In vitro development of pluripotent stem cells is explored as a model of embryogenesis to assess the developmental toxicity of various chemicals

Pluripotent stem cells are derived from the epiblast, a pluripotent tissue in the pre‐ and peri‐implantation stage embryo, or from somatic cells that have been reprogrammed with the Yamanaka factors (Martello & Smith, 2014; Takahashi & Yamanaka, 2016; Figure 1). The former is commonly referred to as embryonic stem (ES) cells, while the latter as induced pluripotent stem (iPS) cells. ES and iPS cells are maintained in the culture dish as undifferentiated cell populations, while retaining the property of pluripotency, that is, the ability to give rise to derivatives of all three germ layers, namely, ectoderm (e.g., neuron), mesoderm (e.g., cardiomyocyte), and endoderm (e.g., hepatocyte). Pluripotent stem cells can be induced under special culture conditions to differentiate into various types of cells, including neurons and cardiomyocytes (Figure 1). The basic idea behind in vitro assays using pluripotent stem cells is to define the inhibitory impact of a chemical exposure on the differentiation as a sign of developmental toxicity. The first developmental toxicity assay, reported as the embryonic stem cell test (EST), employed the differentiation of contracting cardiomyocytes from mouse ES cells as an in vitro embryogenesis model to test the impact of chemicals (Laschinski, Vogel, & Spielmann, 1991; Riebeling et al., 2012; Seiler, Buesen, Visan, & Spielmann, 2006). Since then, many versions of developmental toxicity assays have been reported, which employed the differentiation of ES and iPS cells of mouse and human into various cell types, including neurons and osteoblasts (Kim, Che, & Yun, 2019; Luz & Tokar, 2018; Mennen, Oldenburger, & Piersma, 2022). These assays exploit a variety of endpoint analyses using morphological and molecular techniques to measure the adverse impact on cell differentiation. Such alterations and diversifications of the methodologies are intended to improve the sensitivity and specificity to detect various types of chemicals, and also to streamline the assay procedure to enable high‐throughput analyses of numerous chemicals in a short period of time.

In recent years, pluripotent stem cells are also used to generate “organoids” to recapitulate the more complex three‐dimensional (3D) architectures of tissues and organs in vitro. Organoids are comprised of multiple cell types that are arranged in a spatially organized manner, mimicking the morphology and function of the corresponding organs, such as the intestine, kidney, and brain (Lancaster & Knoblich, 2014; Sasai, 2013). The stem cell‐derived organoids have been explored as in vitro models of the organs in toxicology research (Matsui & Shinozawa, 2021; Wang et al., 2021). “Gastruloids” refer to the 3D aggregates of pluripotent stem cells that mimic the key aspects of gastrulation, namely axial patterning and elongation morphogenesis (Baillie‐Benson, Moris, & Martinez Arias, 2020; van den Brink & van Oudenaarden, 2021; Figure 1). Because gastrulation is the critical embryological process to construct the basic body plan, gastruloids have been used as in vitro embryogenesis models to test the developmental toxicity of chemical exposures (Mantziou et al., 2021; Marikawa, Chen, Menor, Deng, & Alarcon, 2020; Warkus & Marikawa, 2017; Warkus, Yuen, Lau, & Marikawa, 2016). Whether based on the differentiation of a single cell type or the morphogenesis of gastruloids, most of the reported assays are allegedly effective in detecting various chemical exposures that are known to be developmentally toxic. Therefore, the use of pluripotent stem cells for predictive developmental toxicity screening appears to be warranted.

Nonetheless, assays using pluripotent stem cells have not yet become the gold standard for developmental toxicity screening. No regulatory agency mandates the use of stem cell‐based tests to assess the adverse impact of new chemicals on embryogenesis. Although the recent International Council for Harmonization (ICH) Guideline on Detection of Toxicity to Reproduction for Human Pharmaceuticals recommends the use of in vitro assays for developmental toxicity assessment (ICH, 2020), it does not endorse any specific method, including those employing pluripotent stem cells. Does this mean that none of the stem cell‐based assays is satisfactory? If so, what are their deficiencies? Among those reported assays, which ones are better than the others? Can they even be compared with each other? Currently, there is no clear consensus among research communities on how to measure the efficacy or accuracy of individual developmental toxicity assays in a comparable manner. Thus, it is difficult to evaluate objectively which versions of assays are more effective than others. This situation discourages stakeholders, including regulatory agencies and chemical industries, to adopt any specific stem cell‐based assays as part of the decision‐making process in developmental toxicity assessment. Until these issues are resolved, pluripotent stem cells may not become the cornerstone in our collective efforts to identify chemicals that are harmful to embryos.

In this article, several key issues are discussed regarding developmental toxicity assays using pluripotent stem cells, including their need for toxicity screening in addition to other types of alternative assays, strategies to validate and select stem cell‐based assays, and limitations that are inherent to stem cell‐based assays. It is my sincere hope that these discussions would help improve and invigorate stem cell‐based assays to be widely accepted as the gold standard in predictive risk assessment of chemical exposures for developmental toxicity. Note that in this article, the term “developmental toxicity” is mainly used to describe any insult that disturbs embryogenesis to cause death or malformations. It broadly corresponds to “teratogenicity” or “embryotoxicity,” which are also used in many literatures to describe insults on embryogenesis. Also, the term “stem cells” used in this article specifically refers to “pluripotent stem cells” for convenience, and does not include tissue‐ or organ‐specific stem cells that do not possess the pluripotency.

## 2 THE NEED FOR STEM CELL MODELS

Although pluripotent stem cells have the potential to become any cell type, their in vitro development recapitulates only a small fraction of the entire embryogenesis. This is certainly the case for various stem cell models that have been used for developmental toxicity assays, including cardiomyocyte differentiation and gastruloid morphogenesis. If stem cell models are such incomplete representations of the actual embryo, why are they needed for developmental toxicity assessment? Generally, “models” are used in various fields of biomedical research, including developmental toxicology, for two reasons. One is due to ethical issues. The conduct of experimentations involving human subjects poses a variety of ethical concerns. Clinical trials of pharmaceuticals usually exclude women that are pregnant or are planning to conceive, due to the potential adverse impact on unborn children. Even experimentations with human embryos obtained from the surplus of in vitro fertilization procedures or from elective abortions are highly controversial (Lee, Feeney, Schmainda, Sherley, & Prentice, 2020; Wertz, 2002). Thus, laboratory animals are typically used for experimentations to assess the developmental toxicity of chemicals. However, the sacrifice of a large number of animals for toxicity testing is also a contentious ethical issue. There are great interest and demand among the research and regulatory communities in moving toward nonanimal platforms for toxicity assessment. To circumvent such ethical issues associated with human‐ and animal‐based tests, pluripotent stem cells may be needed as models of embryogenesis even though they are incomplete representations.

The other reason to employ models is for practicality. Embryogenesis is an extremely complex process. The fertilized egg gives rise to diverse arrays of tissues and organs in a spatially and temporally dynamic manner. Mammalian embryos, in particular, develop inside of the mother with physical and physiological association through the placenta. Such complexity often hinders mechanistic investigations into how a chemical exposure disturbs embryogenesis. Stem cell‐based models represent specific structures of the embryo at specific developmental stages. There is no maternal environment that may significantly alter the property and amount of chemicals. Due to the in vitro nature, stem cell models are more accessible to a plethora of experimental interrogations in a controlled fashion. Furthermore, assays using pluripotent stem cells are generally faster, cheaper, less labor‐intensive, and more scalable for high‐throughput analyses than animal‐based assays. For these practical reasons, stem cell assays may be needed to obtain the information on developmental toxicity for a large number of chemicals in a speedy manner, and also to elucidate the molecular mechanisms underlying the developmental toxicity of chemical exposures.

Other nonmammalian organisms are also explored as alternatives in developmental toxicity assessment, such as fish, frog, nematode, and slime mold (Baines, Wolton, & Thompson, 2021; Beekhuijzen et al., 2015; Boyd, Smith, & Freedman, 2012; Song et al., 2004). These whole organism systems are likely to bring about valuable insights into the adverse actions of various chemical exposures. Nonetheless, they are evolutionarily distant from mammals, and their gene sequences (i.e., protein primary structures) are considerably different, which may influence the susceptibility to some chemical exposures. Also, mammals have adopted the viviparous mode of reproduction, in which embryogenesis depends on the nutrients and other chemical building blocks that are provided through the mother. By contrast, for nonmammalian organisms, those essential chemicals are already stored inside of the oocyte, and are sufficient to support the entire embryogenesis. Such fundamental disparities in the mode of chemical usage may contribute to the significant differences in the impact of chemical exposures. For these reasons, assays involving pluripotent stem cells are needed to reflect the unique nature of mammalian embryogenesis.

## 3 VALIDATION OF STEM CELL MODELS

Diverse versions of assays have been reported, using different stem cell lines that are differentiated into different cell types or structures. The impact of chemical exposures is also measured differently using various methods, involving specific morphological or molecular features that are linked to the differentiation. Every single one of these assays appears to exhibit fairly good performance according to the validation criteria employed in each study. The question is, can these assays be ranked according to the reported concordance rates (%) to determine which assay is the most effective? The answer is no, mainly because most of these assays were validated differently, that is, using different combinations of reference chemicals with different criteria to define adverse effects. To enable fair comparisons between the different assays, they need to be validated using a common set of reference chemicals. Such a standardized method of validation should reveal the strengths and weaknesses of individual assays in a comparable manner, and help identify which assays are more suited (or not suited) to detect certain types of developmental toxicity.

However, the choosing of reference chemicals for assay validation has been a highly challenging task with a long history of struggles (Brown, 2002; Daston et al., 2010; Marx‐Stoelting et al., 2009; Smith et al., 1983; Webster, Brown‐Woodman, & Ritchie,^1997^). It requires understanding of the principles of teratology (developmental toxicology) as well as a tremendous level of cooperation and coordination among researchers and regulatory bodies. Below, some of the key issues concerning reference chemicals are discussed to help navigate our concerted efforts to establish effective validation strategies.

### 3.1 Exposure‐based validation

“The dose makes the poison” is perhaps the most important dogma in toxicology, often credited to Paracelsus (Grandjean, 2016). Essentially, any chemical can be toxic at a high enough concentration, but it can also be safe at a low enough concentration. This is certainly the case for developmental toxicity as well. In many studies, assay validations were performed using reference chemicals that are categorized as either “nonembryotoxic” or “embryotoxic,” the latter of which may be further divided into multiple groups, such as “weakly embryotoxic” and “strongly embryotoxic” (Genschow et al., 2002; Marx‐Stoelting et al., 2009). However, even those chemicals that are categorized as strongly embryotoxic can be safe when concentrations are low enough. Thus, validation of assays using reference chemicals should be performed with great attention to their concentrations.

Daston and colleagues have pointed out this matter most eloquently (Daston et al., 2010), and generated a list of reference “exposures,” each of which is a specific concentration of a specific chemical, designated either as developmentally toxic or nontoxic exposure (Daston et al., 2014). These exposures are selected based on in vivo rat studies, where adequate toxicokinetic data are available, namely maternal plasma maximum concentration *C*_max_ and the incidence of embryonic death or malformations. The list, often dubbed as the Daston list, may be used to validate the effectiveness of stem cell‐based assays. An effective assay is expected to show an adverse reaction in response to a chemical at the developmentally toxic concentration but not at the nontoxic concentration. Such exposure‐based validation is in line with the principles of toxicology, and allows the evaluation of the sensitivity and specificity of individual assays in a manner relevant to “the real world of developmental toxicity” (Daston et al., 2014). Thus far, unfortunately, only a few assays have been evaluated using the Daston list (Cassar et al., 2019; Marikawa, Chen, Menor, Deng, & Alarcon, 2020; Warkus & Marikawa, 2017).

**FIGURE 2:**
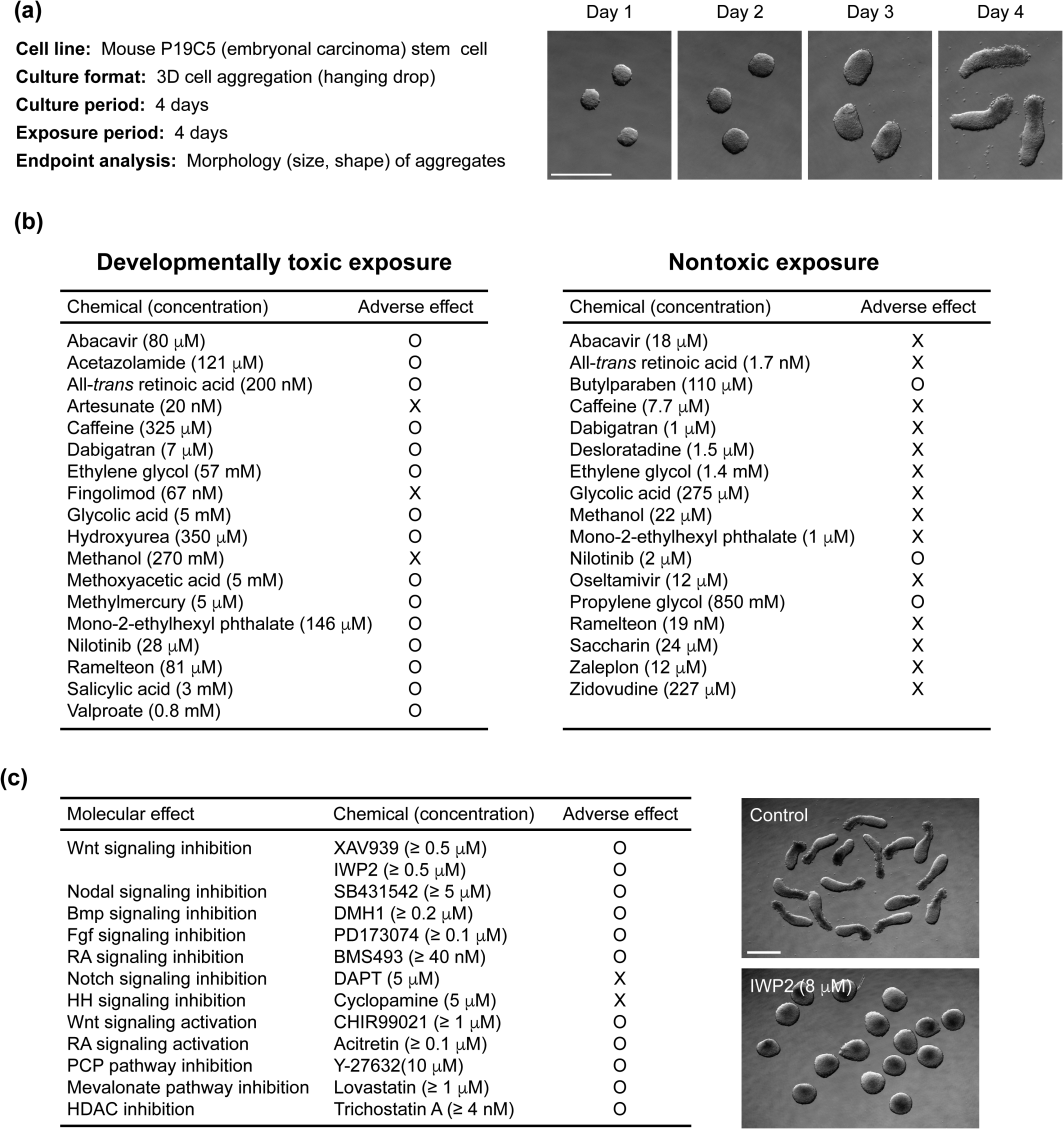
Developmental toxicity assay using gastruloids of mouse P19C5 stem cells. (a) Basic characteristics of the P19C5 gastruloid‐based assay. Right images show the morphological changes of 3D cell aggregates of P19C5 stem cells over the course of 4 days of hanging drop culture. Aggregates have been removed from hanging drops for photography. Scale bar = 500 μm. (b) Exposure‐based validation of the P19C5 gastruloid assay using the Daston list (Daston et al., 2014). Summarized results from the previous studies (Li & Marikawa, 2016; Warkus & Marikawa, 2017) are shown. For adverse effects, circles represent significant morphological impact (i.e., reduced size or altered shape in Day 4 aggregates), and crosses represent no significant morphological impact. (c) Mechanism‐based validation of the P19C5 gastruloid assay. Summarized results from the previous studies (Li & Marikawa, 2015, 2016; Warkus, Yuen, Lau, & Marikawa, 2016; Warkus & Marikawa, 2018) are shown. RA (retinoic acid), HH (hedgehog), PCP (planar cell polarity), HDAC (histone deacetylase). Right images show Wnt signaling‐inhibited Day 4 aggregates (treated with IWP2 at 8 μM) and the corresponding control (untreated) aggregates. Note that axial elongation is markedly diminished in IWP2‐treated aggregates. Scale bar = 500 μm

The results of one of the assays that have been evaluated with the Daston list are summarized in Figure 2. The assay is based on the growth and morphogenesis of gastruloids that are made of 3D aggregates of mouse P19C5 stem cells (Lau & Marikawa, 2014). P19C5 cell aggregates grow and elongate with a distinct anterior–posterior axis during 4 days of culture, recapitulating the morphogenetic process of gastrulation (Figure 2a). In this assay, cell aggregates are treated with test chemicals at various concentrations throughout the entire culture period, followed by morphometric measurement of the size and shape of individual aggregates. A significant reduction in size or a significant alteration in shape in comparison to the corresponding control (untreated) gastruloids is defined as the adverse impact of a chemical exposure. Overall, 29 out of the 35 exposures tested (82.9%) are found to affect P19C5 gastruloids in a manner consistent with the in vivo developmental toxicity data (Li & Marikawa, 2016; Warkus & Marikawa, 2017; Figure 2b). However, this also indicates that the assay is not effective for six specific exposures. Namely, three developmentally toxic exposures (artesunate at 20 nM, fingolimod at 67 nM, and methanol at 270 mM) are “false negative,” whereas three nontoxic exposures (butylparaben at 110 μM, nilotinib at 2 μM, and propylene glycol at 850 mM) are “false positive.” There may be specific reasons why the assay is insensitive or over‐sensitive to these six exposures, as discussed previously (Warkus & Marikawa, 2017). Regardless, this exposure‐based validation has provided critical information on the sensitivity and specificity of the P19C5 gastruloid assay, which may be compared with other assays (once evaluated with the Daston list) to underscore their strengths and weaknesses.

The Daston list can be expanded further to validate developmental toxicity assays more extensively. The current list is restricted to exposures that are based on rat studies. This is because rats are the most commonly used species in developmental toxicity testing. However, as the major goal is to predict human developmental toxicity, it is ideal to include chemical exposures that are known to disturb human embryogenesis. Even though human toxicokinetic studies are limited, there may be sufficient information on the plasma concentrations and developmental toxicity for certain pharmaceutical drugs through clinical and epidemiologic studies. The recent ICH Guideline lists a series of reference chemicals to be used for alternative (in vitro, ex vivo, and nonmammalian in vivo) assays (ICH, 2020). Some of the chemicals are listed as “human teratogens” and are provided with the information on human plasma *C*_max_. The Guideline also provides the no‐observed‐adverse‐effect level (NOAEL) *C*_max_ and/or the lowest‐observed‐adverse‐effect level (LOAEL) *C*_max_ for several chemicals based on mouse, rat, or rabbit studies. These reference chemicals with specific concentrations, including the human teratogens, may be combined with the Daston list to augment the exposure‐based validation.

### 3.2 Mechanism‐based validation

Embryogenesis is characterized by the highly elaborate regulation of cellular behaviors, such as proliferation, differentiation, and migration, which create various tissues and organs in the right place at the right time. These cellular behaviors are genetically controlled and are dependent on the activities of distinct molecular machineries, including signal transduction pathways and transcription factors. In the past several decades, experimental studies using the laboratory mouse as well as other nonmammalian organisms have greatly expanded our knowledge of the molecular machineries that are essential for embryogenesis. In principle, any of these machineries can potentially be targeted by chemical exposures to cause embryonic death or malformations. Thus, another way to validate the effectiveness of developmental toxicity assays is to examine whether they are capable of detecting disturbances in the molecular machineries essential for embryogenesis.

Several core signaling pathways, namely Wnt, Nodal, Bmp, Fgf, Notch, Hedgehog, and Retinoic acid (RA), operate at multiple places and times in the embryo to control distinct cellular behaviors. For example, RA signaling regulates the patterning and morphogenesis of the neural tube, paraxial mesoderm, pharyngeal arches, facial prominences, limbs, heart, and vascular system (Knudsen, Pierro, & Baker, 2021; Piersma, Hessel, & Staal, 2017). Accordingly, a disturbance in RA signaling during embryogenesis, through its inhibition or excessive activation, can cause abnormalities in these structures. Figure 2c summarizes the performance of the P19C5 gastruloid assay in response to the pharmacological inhibitors of the core signaling pathways (Li & Marikawa, 2015). The size and shape of gastruloids are significantly altered by the inhibitors of the Wnt, Nodal, Bmp, Fgf, and RA signaling pathways, indicating that the gastruloid morphogenesis is controlled by these molecular machineries. However, inhibition of Notch or Hedgehog signaling does not cause significant changes in the morphology of gastruloids. Notch signaling is active during gastruloid development, and the pharmacological inhibitor reduces the expression levels of the Notch signaling target genes (Li & Marikawa, 2015). Nonetheless, the growth and elongation of gastruloids appear to be independent of Notch signaling. By contrast, Hedgehog signaling is apparently inactive during gastruloid development, and the effect of the inhibitor (cyclopamine) is undetectable by either morphological or molecular analyses (Li & Marikawa, 2015). Overall, the mechanism‐based validation has provided mechanistic insights into the capability of the gastruloid assay. Namely, it can detect chemical exposures that interfere with Wnt, Nodal, Bmp, Fgf, and RA signaling, but not those that inhibit Notch or Hedgehog signaling.

Clearly, embryogenesis is regulated by many more molecular machineries than the seven core signaling pathways mentioned above. Figure 2c also shows the impact of a few additional chemical exposures on the P19C5 gastruloid, which have different mechanisms of actions (Li & Marikawa, 2016; Warkus & Marikawa, 2018). Examination of more machineries should further illuminate on the capability and limitation of the assay. The mechanism‐based validation described here is akin to the adverse outcome pathway (AOP) framework, which uses the available mechanistic information on a toxicological response to describe linkages between molecular events and adverse outcomes (Draskau, Spiller, Boberg, Bowles, & Svingen, 2020; Rogers, 2020). The AOP‐ or mechanism‐based approach integrates the information from developmental biology and pharmacology, and should help transform developmental toxicology from an empirical to a more predictive science. One of the skeptical opinions over stem cell‐based assays is that their representations of embryogenesis (e.g., cardiomyocyte differentiation or gastrulation) are too narrow to detect a wide range of developmentally toxic chemicals. However, various processes of embryogenesis are controlled by finite sets of molecular machineries, such as the core signaling pathways. Thus, a stem cell model that recapitulates only a limited process of embryogenesis may be able to detect chemical exposures that affect other embryological processes that are not directly represented by the model.

### 3.3 Selection of assays

**FIGURE 3:**
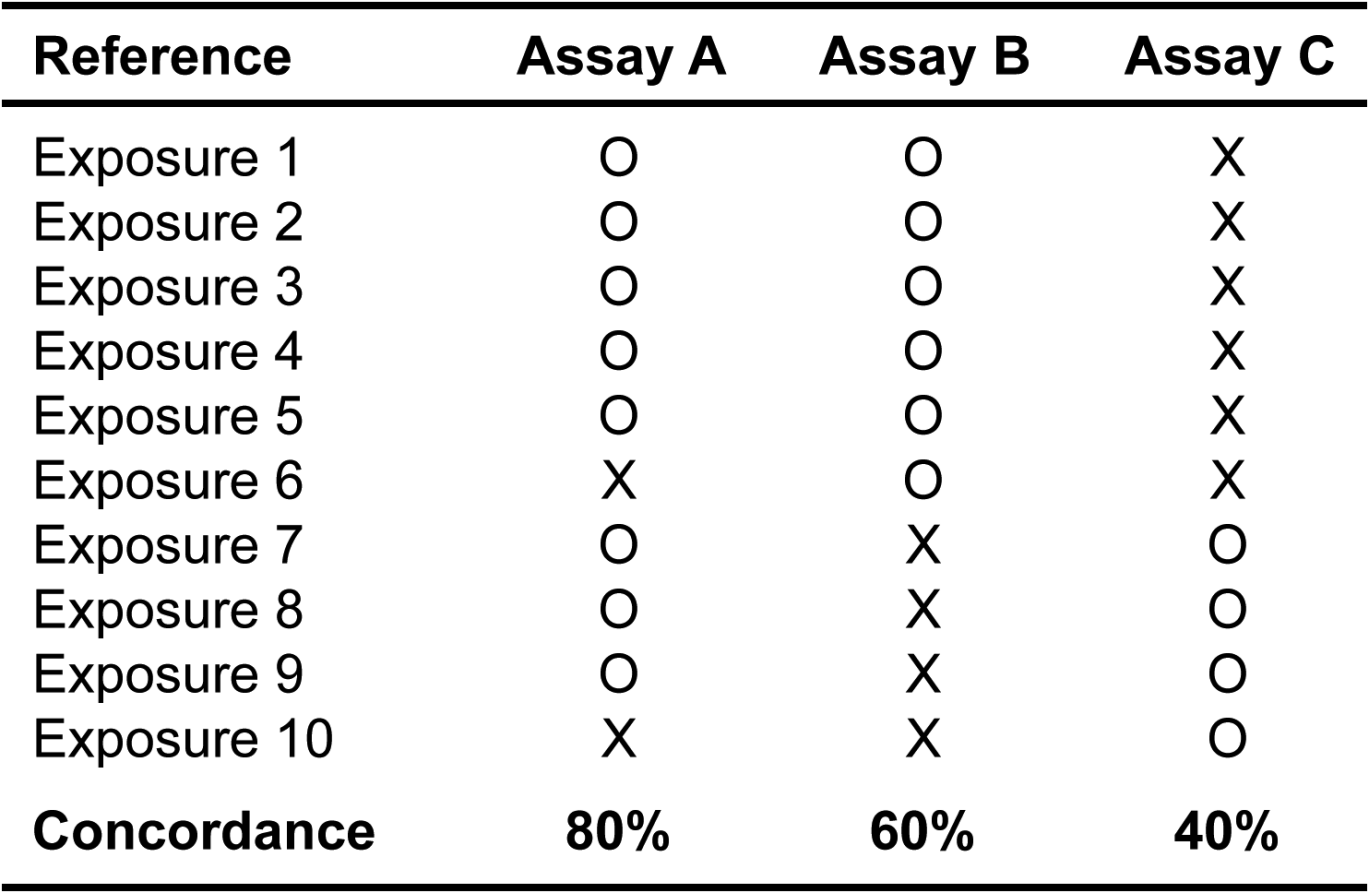
Comparisons of three hypothetical assays that are validated against 10 hypothetical reference (developmentally toxic) exposures. See the text for details

Once evaluated using standardized reference lists, different assays can be objectively compared with each other to reveal their strengths and weaknesses. Only through such fair comparison that the most useful assays may be selected for developmental toxicity screening. Importantly, assays with the highest concordance rates (%) may not necessarily be the most useful ones. This notion is exemplified in the following hypothetical scenario. For 10 toxic reference chemical exposures, assays A, B, and C exhibit the detection capability of 80% (8/10), 60% (6/10), and 40% (4/10), respectively (Figure 3). Intuitively, assay A with 80% concordance may seem to be most useful. However, when looked at closely, a combination of assays B and C yields 100% concordance, which is better than assay A alone or its combination with B or C.

A single type of assay, which is based on a limited representation of embryogenesis, would not be able to detect a whole range of chemicals with various mechanisms of actions that target different embryological processes. However, carefully selected multiple types of assays may be able to detect much more diverse chemicals by complementing the weaknesses of each other. Such selection is only possible when individual assays are evaluated using standardized reference lists. While actual selection must await the results of validations, it may probably require a combination of assays that involve different stem cell lines of different species (e.g., mouse and human), different representations of embryogenesis (e.g., cell differentiation and morphogenesis), and different endpoints of analyses (e.g., morphology and gene expression profile).

Certain practical aspects may also need to be taken into consideration for the selection of assays, if possible. This is because multiple types of assays are to be performed by various investigators. For example, the assay procedures should be relatively easy to conduct by reasonably trained personnel in a consistent manner. The stem cell lines used in the assays should be widely available or freely shared, and the necessary reagents and equipment should be reasonably priced and obtainable.

## 4 OVERCOMING THE LIMITATIONS OF STEM CELL MODELS

In a stem cell‐based assay, the in vitro development of stem cells is used as a model of embryogenesis to assess the adverse effects of chemical exposures. However, there are crucial differences between embryogenesis in vitro and in vivo (i.e., in the mother), which may pose limitations to the application of stem cell models. Some of such limitations are described below to discuss how they may influence stem cell‐based assessment and how they could be overcome.

### 4.1 Differences in the surrounding environment

In vivo, the embryo is exposed to the amniotic fluid, which has distinct compositions of organic and inorganic substances that dynamically change over the course of development (Tong et al., 2009; Underwood, Gilbert, & Sherman, 2005). In addition, once the placenta and the embryonic cardiovascular system are established, substances in the maternal circulation are transported into the embryonic circulation, some are passively while others are selectively (Brett, Ferraro, Yockell‐Lelievre, Gruslin, & Adamo, 2014). Thus, embryonic tissues in vivo are surrounded by materials that are significantly different in type and amount from the culture medium to which stem cells are exposed. The culture media are usually augmented with an excess amount of basic nutrients (e.g., glucose, amino acids), while they are deficient in a complex array of various macromolecules (e.g., proteins, lipids). The differences in the surrounding environment may potentially influence how a given chemical exposure exerts developmental toxicity.

Protein binding is one of such differences between the in vivo and in vitro environments that may affect the actions of chemicals. Certain chemicals, including pharmaceutical drugs, have a high binding affinity to serum proteins, namely albumin. Generally, only unbound forms can diffuse freely and contribute to their pharmacological actions (Smith, Di, & Kerns, 2010). The level of the serum protein (albumin) is typically much lower in the stem cell culture medium than in the plasma. Thus, even when the total concentration (i.e., protein‐bound and unbound forms) of a test chemical is comparable between the plasma and the culture medium, the concentration of the unbound form may be higher in the latter to exert more potent effects. As a result, stem cell‐based assays may misleadingly appear “over‐sensitive” to certain chemicals, especially those with an extremely high affinity to albumin. Depending on the binding property of test chemicals and the serum protein concentration in the culture medium used, the exposure‐based validation of stem cell assays (discussed in Section 3.1.) may need to be calibrated accordingly.

### 4.2 Maternal influences

In vivo, the embryo is under the influence of the mother, which is absent in stem cell models. Maternal influences can significantly affect whether and how chemicals disturb embryogenesis. For example, certain chemicals are known as proteratogens, which by themselves are nontoxic but become developmentally toxic after metabolic conversions by the mother, mainly in the liver (Wells & Winn, 1996). Stem cell‐based assays, as well as other in vitro and ex vivo assays, may be unable to detect proteratogens, as they lack maternal metabolisms. To overcome such limitation, several attempts have been made to augment in vitro or ex vivo assays by incorporating exogenous metabolic systems, such as hepatocytes or liver extracts, to simulate the effects of the maternal liver (Hettwer et al., 2010; Luijten, Verhoef, Westerman, & Piersma, 2008; Oglesby, Ebron, Beyer, Carver, & Kavlock, 1986; Ozolins, Oglesby, Wiley, & Wells, 1995; Piersma et al., 1991; Zhao, Krafft, Terlouw, & Bechter, 1993), although it is unclear how effectively such liver substitutes can represent the complexity of maternal metabolisms. For many pharmaceutical drugs, the information on their metabolisms in the human body is often available through pharmacokinetic studies, including their chemical structures and the plasma concentrations of the major metabolites. The testing of individual metabolites using stem cell assays, which would be much easier to do with in vitro systems, may reveal that the parent compounds can act as proteratogens.

Some chemicals may cause developmental toxicity indirectly through the mother. For example, embryogenesis can be severely disturbed when chemical exposures interfere with the function of the uterus by blocking the blood supply or the endometrial maintenance. Chemicals that cause hyperthermia or hypoglycemia in the mother may also adversely impact embryogenesis indirectly (DeSesso, 1987; Lancaster, 2011; Persson & Hansson, 1993). Such chemicals may not be detectable with stem cell assays nor with any other alternative methods (in vitro, ex vivo, and nonmammalian in vivo).

## 5 FUTURE PERSPECTIVES

Our collective efforts are in progress, aiming to transform stem cell‐based assays into the gold standard in predictive developmental toxicity assessment, even though they cannot entirely replace human‐ or animal‐based studies. As discussed above, stem cell models have several inherent limitations and may not detect certain types of chemicals that cause developmental toxicity in vivo. The absence of an adverse effect in stem cell‐based assays does not guarantee the safety of the chemical exposures examined. Those exposures need to undergo additional in vivo studies, including animal experimentations, to assure their safety. By contrast, with the use of well‐validated and well‐selected stem cell assays (as discussed in this article), the presence of an adverse effect can be regarded as a “red flag.” Ideally, the data obtained from stem cell assays should be reliable enough to define adverse chemical exposures as developmentally toxic, and to regulate them accordingly. For example, no additional animal test should be required or permitted to evaluate their developmental toxicity in vivo, unless the necessity to do so is scientifically justified. For pharmaceutical drugs, they should be labeled with a pregnancy risk warning. In the case of industrial compounds, their marketing or release to the environment should be restricted by regulatory agencies.

To achieve such an ambitious goal, the imminent task is to build standardized reference lists of chemical exposures, which are essential to validate individual stem cell assays for comparison and selection. It would require enormous levels of collaboration and conviction among communities of toxicologists and developmental biologists, who can provide their expertise in gathering information that are appropriate for exposure‐based and mechanism‐based validations. It would also be helpful if standardized reference lists are officially endorsed by the Society for Birth Defects Research and Prevention, not only to promote, but also to mandate the usage of the reference lists for the validation of alternative developmental toxicity assays.

Even after the most effective combination of assays is selected, there may still be reluctance or skepticism among research and regulatory communities to accept stem cell‐based assays as the gold standard. To build more confidence among skeptics, stronger demonstrations may be necessary to show that stem cell assays can predict the developmental toxicity of new chemicals “before” any in vivo study is performed. For example, a series of new chemicals, such as those that are synthesized during drug development as candidate compounds, may be first tested by stem cell assays for adverse effects. Compounds with adverse effects are then tested in animals to collect toxicokinetics data, namely the plasma concentrations and the incidence of embryonic death or malformations. If the in vivo data are consistent with the results of stem cell assays, then it would unequivocally demonstrate their predictive power. The use of animals for such purposes may appear contradictory to the goal of nonanimal alternative approaches. However, once accepted as the gold standard, stem cell assays can, in the long run, bring down the animal usage significantly. While other nonanimal alternative approaches, including in silico models (Scialli et al., 2018), can also provide valuable and critical insights into the effects of chemical exposures, it is my belief that pluripotent stem cells will play the major role in predictive developmental toxicity assessment.
